# A Comprehensive Analysis of the Effect of A>I(G) RNA-Editing Sites on Genotoxic Drug Response and Progression in Breast Cancer

**DOI:** 10.3390/biomedicines12040728

**Published:** 2024-03-25

**Authors:** Yanara A. Bernal, Alejandro Blanco, Eduardo A. Sagredo, Karen Oróstica, Ivan Alfaro, Katherine Marcelain, Ricardo Armisén

**Affiliations:** 1 Centro de Genética y Genómica, Instituto de Ciencias e Innovación en Medicina (ICIM), Facultad de Medicina Clínica Alemana, Universidad del Desarrollo, Santiago 7610658, Chile; ybernalg@udd.cl (Y.A.B.); ablanco@udd.cl (A.B.); ialfaro@udd.cl (I.A.); 2 Department of Molecular Biosciences, The Wenner-Gren Institute, Stockholm University, Svante Arrhenius väg 20C, SE-106 91 Stockholm, Sweden; sagredo1989@gmail.com; 3 Department of Microbiology, Tumor and Cell Biology, Karolinska Institutet, SE-171 77 Stockholm, Sweden; 4 Science for Life Laboratory, SE-171 65 Solna, Sweden; 5 Instituto de Investigación Interdisciplinaria, Vicerrectoría Académica, Universidad de Talca, Talca 3460000, Chile; korostica09@gmail.com; 6 Departamento de Oncología Básico Clínica, Facultad de Medicina, Universidad de Chile, Santiago 8380453, Chile; kmarcelain@uchile.cl; 7 Centro de Prevención y Control de Cáncer (CECAN), Universidad de Chile, Santiago 8380453, Chile

**Keywords:** breast cancer, RNA editing, RNA-edited sites, ADAR1, drug sensitivity, PARP inhibitors, anthracyclines, alkylating agents, cancer therapy

## Abstract

Dysregulated A>I(G) RNA editing, which is mainly catalyzed by ADAR1 and is a type of post-transcriptional modification, has been linked to cancer. A low response to therapy in breast cancer (BC) is a significant contributor to mortality. However, it remains unclear if there is an association between A>I(G) RNA-edited sites and sensitivity to genotoxic drugs. To address this issue, we employed a stringent bioinformatics approach to identify differentially RNA-edited sites (DESs) associated with low or high sensitivity (FDR 0.1, log2 fold change 2.5) according to the IC_50_ of PARP inhibitors, anthracyclines, and alkylating agents using WGS/RNA-seq data in BC cell lines. We then validated these findings in patients with basal subtype BC. These DESs are mainly located in non-coding regions, but a lesser proportion in coding regions showed predicted deleterious consequences. Notably, some of these DESs are previously reported as oncogenic variants, and in genes related to DNA damage repair, drug metabolism, gene regulation, the cell cycle, and immune response. In patients with BC, we uncovered DESs predominantly in immune response genes, and a subset with a significant association (log-rank test *p* < 0.05) between RNA editing level in *LSR*, *SMPDL3B*, *HTRA4*, and *LL22NC03-80A10.6* genes, and progression-free survival. Our findings provide a landscape of RNA-edited sites that may be involved in drug response mechanisms, highlighting the value of A>I(G) RNA editing in clinical outcomes for BC.

## 1. Introduction

The heterogeneity in response to anti-cancer drugs and the development of therapy resistance in cancer represent a complex condition that generates significantly higher mortality rates worldwide. Several omics data are available and freely accessible, and they have been widely used in precision medicine; however, RNA modifications have not been extensively compared with genomic and transcriptomic approaches as sources of biomarkers and therapeutic targets in cancer [1]. RNA editing is a post-transcriptional process involved in several biological processes of RNA life and their deregulation has been attributed to health conditions including cancer [2]. The most frequent RNA-editing event involves the deamination of adenosine (A) to inosine (I) (A>I), interpreted by cellular machinery as guanosine (G) [3] on double-stranded RNA (dsRNA), which is typically formed through an interaction between repetitive regions, where this type of editing is most frequent, as well as in non-coding regions [4]. This chemical modification of nucleotides is catalyzed by a family of three isoforms of deaminases (ADARs). Among them, ADAR1 and ADAR2 possess an active catalytic domain, whereas ADAR3 does not [5]. This conversion can induce various molecular alterations, including mRNA recoding, splicing alterations, and non-coding RNA modifications that can affect RNA–RNA interactions and RNA–protein interactions [6]. Moreover, long non-coding RNAs (lncRNAs) can alter the secondary structure of mRNA, potentially affecting its interaction with miRNA. Furthermore, RNA editing may interfere with the processing and function of mature miRNA [7], or if the RNA editing occurs in the untranslated region, it can lead to miRNA regulation changes.

RNA editing has emerged as a pertinent focus in cancer. Both high *ADAR1* expression and specific RNA-edited sites are related to phenotypes and clinical outcomes in breast cancer (BC). First, ADAR1 has a role in apoptosis, the cell cycle, proliferation, and the DNA damage response (DDR) by inducing gene instability [8,9]. Also, specific A>I(G) RNA-edited sites have been described that contribute to the development of cancer phenotypes [10,11]. Additionally, the upregulation of *ADAR1* expression in BC tissues may promote cancer progression [11], and women with BC who have a higher expression of *ADAR1* are associated with lower survival than those with lower *ADAR1* expression [8]. However, missense amino acid changes in the coding region may lead to functional consequences, which have been reported to play a potential driver role in cancer and its pathogenesis [12,13].

Specific A>I(G) RNA-edited sites could be involved in drug response [14,15]. Some A>I(G) RNA-edited sites have been associated with sensitivity to anti-cancer drugs [12] and can even affect the metabolism of these drugs. An A>I(G) RNA-edited site in the 3′UTR region of dihydrofolate reductase (*DHFR*), a key enzyme in folate metabolism and a therapeutic target of methotrexate, induces resistance to methotrexate [16]. Also, specific conserved A>I(G)-recoding RNA-edited sites with clinical relevance like AZIN1^S367G^, COG^I635V^, GRIA2^R764G^, and COPA^I164V^ influence the carcinogenic process [13]. These sites have been involved in sensitivity or resistance to some tyrosine kinase inhibitors (TKIs) in BC cell lines [12]. However, no comprehensive characterization has been made of specific and differentially RNA-edited sites linked to conditions or phenotypes related to the sensitivity of BC to drugs such as PARP inhibitors, anthracyclines, and alkylating agents commonly used in the treatment of this disease.

Here, we identified, characterized, and selected differentially A>I(G) RNA-edited sites (DESs) associated with both low and high sensitivity to genotoxic drugs, including PARPis, anthracyclines, and alkylating agents in BC cell lines. Our findings were validated in patients with BC from The Cancer Genome Atlas (TCGA), and uncovered a significative relationship between women’s survival rate and hyper- or hypo-editing of DESs associated with genotoxic drug sensitivity.

## 2. Materials and Methods

### 2.1. Selection of Breast Cancer Cell Lines by Drug Sensitivity

**Figure 1:**
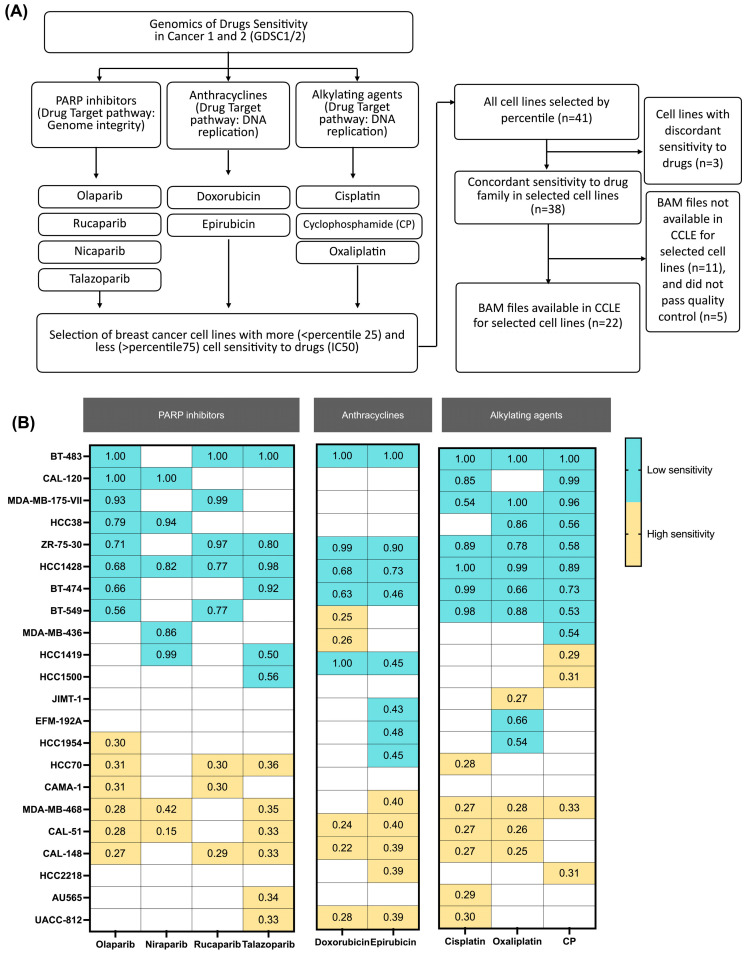
Characterization of global A>I(G) RNA-editing activity and *ADAR1* expression by drug response in selected breast cancer cell lines. (**A**) Flow chart of the selection of breast cancer (BC) cell lines by IC_50_; and (**B**) distribution of IC_50_ normalized in 22 BC cell lines (row) by genotoxic drug (column) with a higher IC_50_ in blue and a lower IC_50_ in yellow.

We considered all BC cell lines (*n* = 51) of the 8.4 release of Genomics of Drug Sensitivity in Cancer 1 (GDSC1) https://www.cancerrxgene.org/ (accessed in August 2022) (Figure 1A). Cell lines were specifically chosen from Genomics of Drugs Sensitivity in Cancer 2 (GDSC2) for PARP inhibitors (olaparib, niraparib, rucaparib, and talazoparib), anthracyclines (epirubicin and doxorubicin), and alkylating agents (cisplatin, oxaliplatin, and cyclophosphamide). The standardized half-maximal inhibitory concentration (IC_50_) was used to classify the BC lines into two groups based on drug sensitivity: those below the 25th percentile (quartile 1) were designated as high-sensitivity cell lines, while those above the 75th percentile (quartile 4) were classified as low-sensitivity cell lines for each drug (Figure 1B). Forty-one BC cell lines (of the total of 51 available) were present for all drugs studied. The BAM files from RNA-seq analyses for 22 BC cell lines were selected and downloaded from the National Cancer Institute GDC data portal https://portal.gdc.cancer.gov/ (downloaded in August 2022). Then, a quality check was conducted for all RNA-seq data using SAMtools stats (v1.6) and the MultiQC program (v1.12) [17,18].

### 2.2. Identification and Characterization of A>I(G) RNA-Edited Sites in Breast Cancer Cell Lines

We employed REDITools2 to identify genomic sites with multiple alleles from RNA-seq BAM files. The command used was: reditools.py -f file.bam -r reference.fasta -S -s 0 -o output_table.tsv, with the reference.fasta corresponding to the hg19 version [19]. Specifically, we focused on A>G RNA editions on the positive strand and T>C editions on the negative strand. Filtering criteria included: total coverage at the called position of at least five edited reads and an allele frequency ≥0.05. After consolidating the lists of edited sites for all cell lines, we utilized the bam-readcount program [20]. This step aimed to determine whether NaN values were caused by low or non-coverage sites or because of a reference/reference position, as REDITools2 was configured to only report edited sites. At the sample level, only the sites present in more than 50% of BC cell lines were used for comparisons. For each cell line, DNA variant calling data was downloaded from DepMapPortal (version 19Q3:hg19). Subsequently, all A/G and T/C editions, depending on their strand, from the RNA-seq dataset were filtered out if they were present in the DepMapPortal dataset, with matching by chromosome, position, and alleles.

### 2.3. Determination of A>I(G) RNA-Editing Level and DESs Selection

The global editing index was represented by the RNA Editing Indexer (available at https://github.com/a2iEditing/RNAEditingIndexer), which was used to represent an initial approach to the global A>I(G) RNA edition with the Alu Editing Index (AEI) [21]. The AEI represents the ratio of the discordant reads at the edited site to the total reads on the site (concordant A/A (positive strand) or T/T (negative strand)) plus discordant (A/G (positive strand) or T/C (negative strand)) from Alu sites [21]. We used the AEI available for all cell lines from the Cancer Cell Line Encyclopedia (CCLE) [22]. To delve deeper into the differentially A>I(G) RNA-edited sites (DESs) by drug sensitivity, REDIT-LLR function, which is a function based on the beta-binomial distribution, was used. The DESs were determined by *p*-value < 0.05, a false discovery rate (FDR) of 0.1 was determined by the Benjamini–Hochberg (BH) correction, and a log fold change of 2.5 was calculated from the ratio of the average RNA-editing level [23]. The average RNA-editing level in the RNA-edited sites was calculated using the maximum likelihood estimate (MLE) derived from the REDIT-LLR output. On examining the association of DESs with low or high sensitivity, a delta of the ratio of coefficients (α/α + β) was calculated for each site. Finally, the RNA-editing level at sites in DESs was determined by the ratio between mismatch (A/G on the positive strand or T/C on the negative strand) reads and total readings at the site (both mismatch and match, represented by A/A on the positive strand or T/T on the negative strand).

### 2.4. Annotation and Prediction of Functional Consequences of RNA-Edited Sites

The Ensembl Variant Effect Predictor (VEP) version 110 was used for annotating and predicting the molecular consequences of DESs, utilizing the hg19 reference genome (GRCh37.p13) [24]. We further refined our results by filtering for one selected consequence per variant allele. To assess the impact of amino acid changes on protein structure and function, predictions from PolyPhen version 2.2.2 and SIFT version 5.2.2 were incorporated. For scoring the deleteriousness of these variants, a Combined Annotation-Dependent Depletion (CADD) score [25] was employed, and we considered a potential splicing alteration when the delta score was >0.5 in SpliceAI prediction (Ensembl/GENCODE v24 canonical transcripts). Distinctive features of the DESs were characterized using information from the VEP output, including strand orientation, biotype, gene symbol, and the specific consequence. Additionally, the RepeatMasker database was utilized to categorize A>I(G) RNA-edited sites based on the type of sequence, distinguishing between Alu elements, non-Alu repetitive elements, and non-repetitive elements [26]. Finally, we explored those DESs related to cancer by cross-referencing the information from the DEG annotation with the Cancer Gene Census (CGC) from the Catalogue of Somatic Mutations in Cancer (COSMIC) V98.

### 2.5. Differential Gene Expression and Global Editing Index by Drug Sensitivity in BC Cell Lines

For studied differences in mRNA ADAR gene expression/global editing index by group, first, we checked for normal distribution with the Shapiro–Wilk test. In the normal distribution of data, we applied a *t*-test; in the non-normal distribution, we applied the Wilcoxon test and considered significative differences when the *p*-value < 0.05. We used a Pearson correlation between mRNA gene expression and the global editing index. For the determined differences in mRNA gene expression in DESs by group, we applied the Wilcoxon test and BH correction, in which we considered the results to be significant when the *p*-adjust value < 0.05. All analyses and plots were constructed using R studio version 2023.06.1+524.

### 2.6. Gene Ontology Analysis in DESs

To delve into functional aspects, gene ontology analysis was performed on the genes associated with DESs using ClusterProfile and the average RNA editing level in each site [27]. Subsequently, we explored differences in mRNA gene expression (log2(TPM+1)) between low- and high-sensitivity BC cell lines, leveraging data obtained from the DepMapPortal.

### 2.7. Validation of DESs in Women with Breast Cancer from TCGA

From a pool of 1084 patients from The Cancer Genome Atlas (TCGA), we selected basal subtype (*n* = 171). Within this subgroup, we employed progression-free survival (PFS) as a criterion to select cases falling within the first and fourth quartiles [28]. Finally, we selected 86 cases. BAM files from RNA-seq were downloaded from the National Cancer Institute GDC data portal https://portal.gdc.cancer.gov (in November 2023). These files, aligned with the hg38 reference genome, underwent quality check analysis. Following the same methodology as detailed previously for cell lines, we employed REDITools2, with reference.fasta corresponding to the hg38 version [19], in which we applied the same filters, total coverage at the called position of at least five edited reads, and an allele frequency ≥0.05. The UCSC tool, LiftOver, was employed to convert the genomic coordinates of DESs in BC cell lines from hg19 (February 2009, GRCh37/hg19) to hg38 (December 2013, GRCh38/hg38), ensuring alignment with DESs in women with BC from TCGA. The average RNA-editing level for each DES was employed for the gene ontology analysis in this cohort [29]. We classified each RNA-edited site found in women as either highly edited or lowly edited when the RNA editing level reached over the mean of the RNA-edited level at each respective site. For survival analysis and the log-rank test, we utilized Survival version 3.5-7, while the Survminer package version 0.4.9 was employed to generate survival curves. We considered differences to be significant when the *p*-value < 0.05 from the log-rank test. For gene expression analysis, we obtained the mRNA gene expression data (RSEM, Batch normalized from Illumina HiSeq_RNASeqV2) of the TCGA Breast Cancer Study from the cBiopPortal [30].

## 3. Results

### 3.1. Characterization of Global A>I(G) RNA-Editing Activity and ADAR1/2 Expression in Low- and High-Sensitivity Breast Cancer Cell Lines

Following the workflow described in the methodology section (Figure 1A), a total of 22 BC cell lines were scrutinized, with 18 dedicated to PARPis (10 low sensitivity and 8 high sensitivity), 15 to anthracyclines (8 low sensitivity and 7 high sensitivity), and 19 to alkylating agents (10 low sensitivity and 9 high sensitivity) (Figure 1B, Table S2).

**Figure 2:**
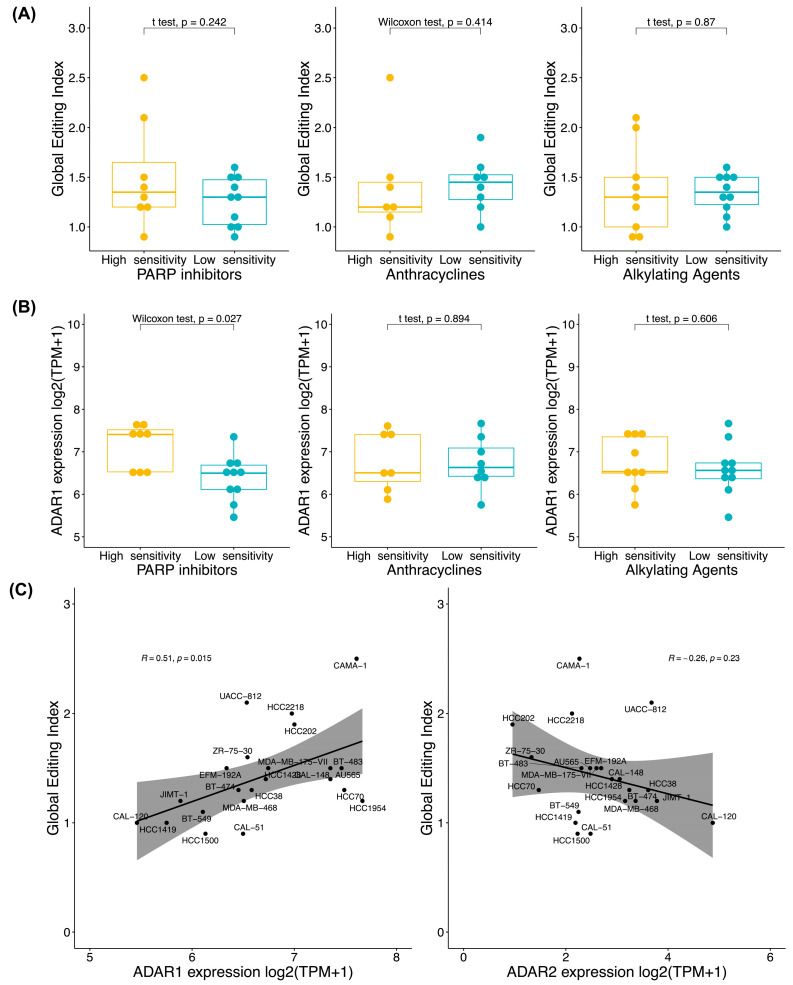
Global A>I(G) RNA-editing activity is partially positively correlated with *ADAR1* expression by drug response in selected breast cancer cell lines. (**A**) Differences in global editing index in BC cell lines by drug sensitivity; (**B**) differences in *ADAR1* expression log2 transcript per million (TPM+1) in BC cell lines by drug sensitivity; and (**C**) Pearson correlation between global editing index and *ADAR1* (left) and *ADAR2* (right) expression log2(TPM+1) in all BC cell lines by sensitivity (high and low) to PARP inhibitors, anthracyclines, and alkylating agents (*n* = 22). We analyzed 18 BC cell lines for PARP inhibitors (low (*n* = 10) and high sensitivity (*n* = 8)), 15 for anthracyclines (low (*n* = 8) and high sensitivity (*n* = 7)), and 19 for alkylating agents (low (*n* = 10) and high sensitivity (*n* = 9)).

For the characterization of ADAR1 and the global editing index of BC cell lines, we initially sought differences between the global editing index and *ADAR1* expression based on drug sensitivity. Notably, no discernible differences were identified in *ADAR1* expression (Figure 2A) or *ADAR2* expression (Figure S1) within any drug families, nor in the global editing index analysis (Figure 2B). Subsequently, we examined whether the global editing index correlated with *ADAR* mRNA gene expression. We observed a moderate positive correlation between the global editing index and *ADAR1* expression in all selected BC cell lines (R = 0.51, *p* < 0.05). However, no correlation was evident with *ADAR2* expression, with a noticeable tendency toward a negative correlation (Figure 2C).

While these findings offer partial insights into ADAR1 activity through the global editing index, our subsequent analysis delved comprehensively into specific RNA-edited sites based on genotoxic drug sensitivity.

### 3.2. Identification of Differentially A>I(G) RNA-Edited Sites in Breast Cancer Cell Lines with Low and High Sensitivity to Genotoxic Drugs

**Figure 3:**
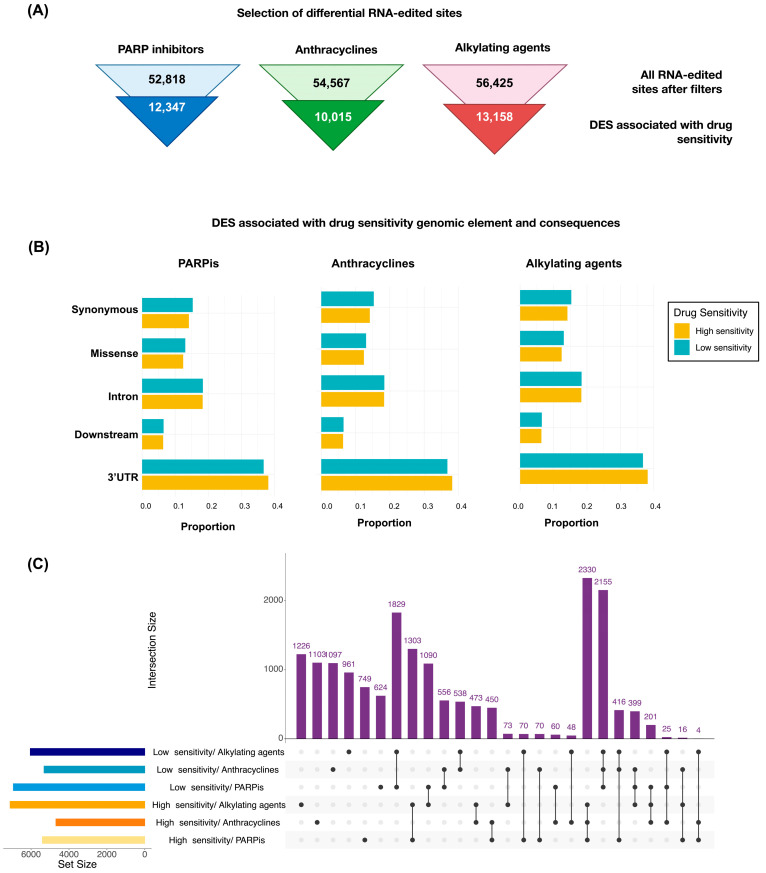
Overview of differentially RNA-edited sites associated with drug sensitivity. (**A**) Synopsis of differentially A>I(G) RNA-edited sites (DESs) associated with drug sensitivity identified in breast cancer (BC) cell lines; (**B**) distribution of DESs through genomic elements and consequences; and (**C**) distribution of number of DESs by sensitivity and drug family.

Following previous methodologies [19,21] for the identification of RNA-edited sites (Figure S2), after filters, we detected 52,818, 54,567, and 56,425 RNA-edited sites. Among these, 12,541, 10,015, and 13,158 were identified as DESs (*p*-value < 0.05; FDR < 0.1; fold change > 2.5) associated with drug sensitivity, that is, low sensitivity or high sensitivity to PARPi, anthracyclines, and alkylating agents, respectively (Figure 3A). Our characterization extended to DESs across genomic elements, predominantly in the 3′UTR, intron, and protein-coding regions with synonymous and missense amino acid changes (Figure 3B), and we characterized DESs in Alu repetitive regions and in other regions (Figure S3). In Figure 3C, the intersection and exclusivity among the DESs based on sensitivity to the three drug families are shown. We reveal a noteworthy overlap between low and high sensitivity across the three drug families. Specifically, 2155 and 2330 DESs were associated with low and high sensitivity, respectively. Also, we found a large number of sites common between alkylating agents and PARPis for low sensitivity (*n* = 1829) and high sensitivity (*n* = 1303) (Figure 3C, Table S3).

**Figure 4:**
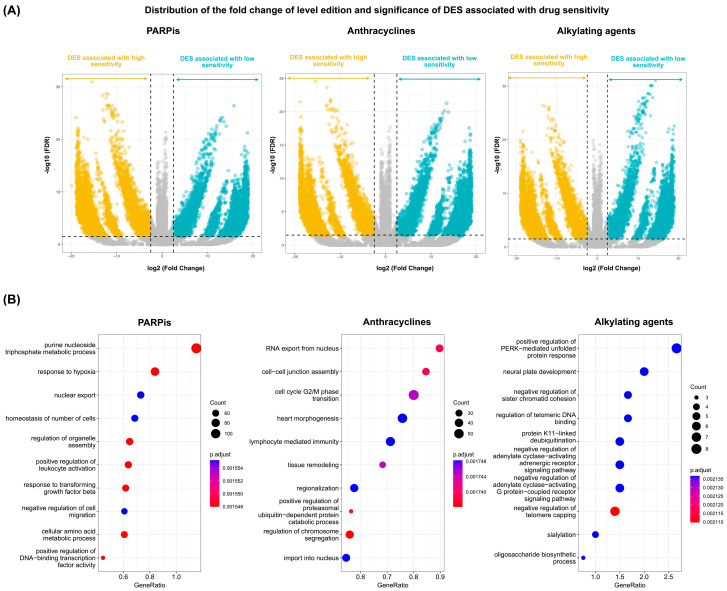
Biological processes enriched in DESs associated with low sensitivity. (**A**) Distribution of DESs according to the fold change of level edition (difference of the ratio of coefficients (*α*/*α* + *β*) from REDITs test) and false discovery rate (FDR), with overedited sites with high sensitivity (yellow) and overedited sites with low sensitivity (blue) to PARP inhibitors, anthracyclines, and alkylating agents; and (**B**) gene ontology analysis of DESs with PARP inhibitors (*n* = 6248), anthracyclines (*n* = 5479), and alkylating agents (*n* = 6473) in the biological process, assessing the proportion of genes associated with each biological function concerning the total number of genes analyzed.

We further detailed their distribution in the log fold change of RNA editing level and false discovery rate (Figure 4A). In the gene ontology analysis, the biological process revealed enriched pathways in DESs associated with drug sensitivity, including the regulation of mRNA processing, RNA splicing, the cell cycle, and the cellular response to stimulus (Figure 4B) in annotated genes (*n* = 6191, *n* = 5478, and *n* = 6472 in PARPis, anthracyclines, and alkylating agents, respectively). We also examined the differential expression of unique genes (*n* = 6853) from differentially edited transcripts (Table S5). We did not find significant differences in the genes where DESs were present. We also provide those variants that could affect splicing (Table S6); however, these are not found in cancer-related genes.

### 3.3. Predicted Pathogenic DESs Associated with Drug Sensitivity in Cancer-Related and Unrelated Genes

We characterized the DESs using predictive tools such as Sorting Intolerant from Tolerant (SIFT) and Polymorphism Phenotyping (PolyPhen), or assessed their clinical significance through ClinVar, designating them as deleterious, probably/damaging, or pathogenic, respectively. Furthermore, our analysis incorporated cross-referencing with the CGC from COSMIC to elucidate their role in cancer. The prioritization focused on pathogenicity favored those DESs in coding regions with amino acid changes. Thirteen prioritized DESs associated with drug sensitivity in transcripts of unrelated cancer genes were manually selected (Table S7) such as RAD51D^E253G^ and TPMT^Y240C^. These findings provide detailed insights into high-confidence DESs with predicted functional consequences, which are associated with low or high sensitivity to genotoxic drugs. Notably, certain DESs are located within oncogenes such as NUP98^T85A^, and tumor suppressor genes such as TP53^Y220^/TP53^Y163C^, BRCA1^Q356R^, ERCC4^E875G^, and KMT2D^D3419G^ (Table S8). Importantly, these findings, uncovered in BC cell lines, lay the groundwork for subsequent investigations to determine the reproducibility of these discoveries in datasets comprising BC tumors.

### 3.4. RNA-Editing Level in DESs Selected in Women’s Basal Breast Cancer

**Figure 5:**
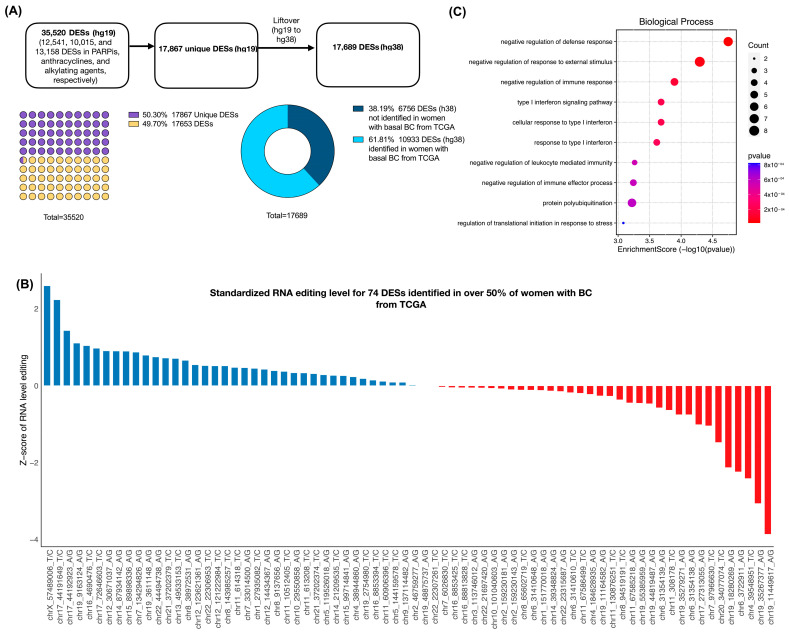
RNA-edited sites associated with drug sensitivity from cell line data discovered in samples from patients with BC from The Cancer Genome Atlas. (**A**) Synopsis of DESs associated with drug sensitivity from cell lines and samples from patients with BC; (**B**) standardized average RNA-editing level in 74 DESs found in women with BC; and (**C**) the biological process from gene ontology analysis of the 74 DESs (66 genes).

Our results were validated in a cohort of 86 women with basal BC from TCGA, grouped according to months of PFS. In these two groups, clinical characteristics are similar (Table S9). In LiftOver, 17,694 sites were converted and 173 failed (Figure 5A). Of 17,689, 10,933 (61.8%) were identified in this cohort, but only 74 DESs (0.67%) were in more than 50% of patients. For this, we show each site’s standardized RNA-edited level (Figure 5B), according to drug classification from cell lines (Figure S4). Additionally, the immune process is highlighted under the biological process from the gene ontology analysis of 66 genes of these DESs (Figure 5C).

**Figure 6:**
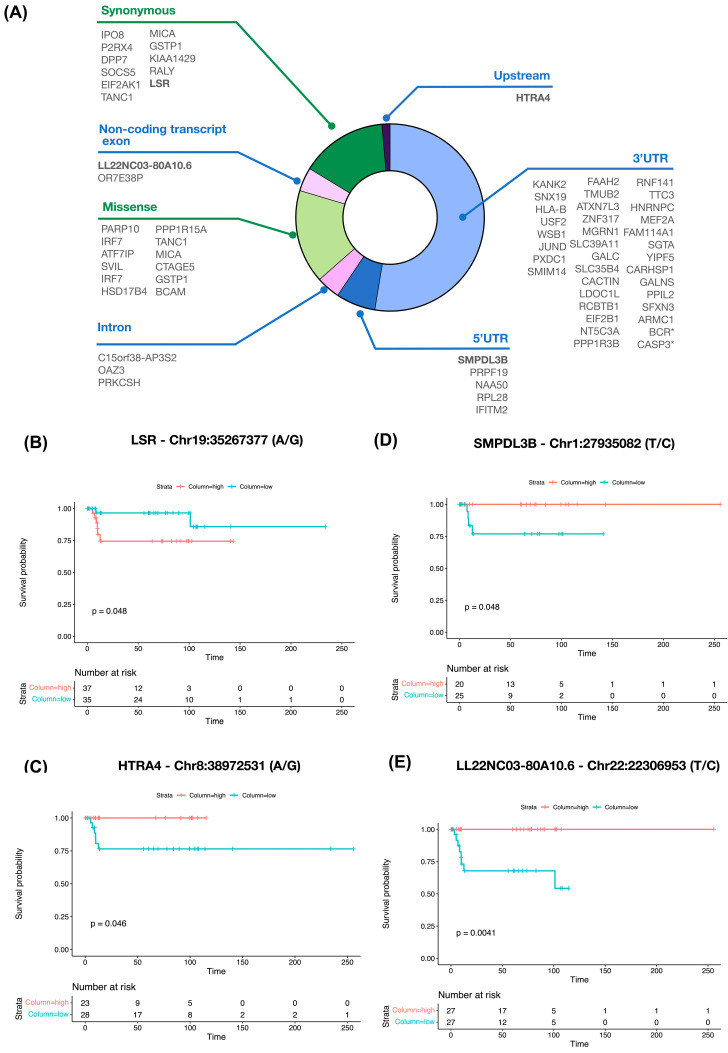
RNA-edited sites associated with drug sensitivity with clinical significance in patients with BC. (**A**) Genomic elements of 74 DESs; in bold are the editing sites associated with progression-free survival and asterisks (*) denote the cancer-related genes according to the CGC of COSMIC; (**B**) a significant relationship between progression-free survival in women with hyper-edited (*n* = 37) DESs was higher than in women with hypo-edited (*n* = 35) DESs in *LSR* (log-rank test *p* < 0.05); and (**C**) in women with hyper-edited (*n* = 23) DESs than in women with hypo-edited (*n* = 28) DESs in *HTRA4* (log-rank test *p* < 0.05); (**D**) a significant relationship between progression-free survival in women with hyper-edited (*n* = 20) DESs than in women with hypo-edited (*n* = 25) DESs was found in *SMPDL3B* (log-rank test *p* < 0.05); and (**E**) a significant relationship between progression-free survival in women with hyper-edited (*n* = 27) DESs than in women with hypo-edited (*n* = 27) DESs was found in *LL22NC03-80A10.6* (log-rank test *p* < 0.05).

Finally, from 74 DESs in each genomic element (Figure 6A), we found a significant relationship between women’s survival rate and DES-editing level (Table S10). Poor survival with the hyper-editing level of a DES on lipolysis-stimulated lipoprotein receptor (*LSR*) (*n* = 72; log-rank test *p*-value = 0.048) was associated with low sensitivity to PARP inhibitors (Figure 6B). The hypo-editing of a DES on HtrA serine peptidase 4 (*HTRA4*) (*n* = 51; log-rank test *p*-value = 0.046) was linked to high sensitivity to anthracycline (Figure 6C), on sphingomyelin phosphodiesterase acid-like 3B (*SMPDL3B*) (*n* = 45; log-rank test *p*-value = 0.048) was associated with low sensitivity to anthracyclines (Figure 6D), and on *LL22NC03-80A10.6* (*n* = 54; log-rank test *p*-value = 0.0041) was associated with low sensitivity to anthracyclines (Figure 6E).

Our discovery of DESs associated with drug sensitivity and carrying functionally harmful consequences, based on cell line data, has illuminated the potential involvement of RNA-edited sites on drug response even beyond the gene expression in our dataset, and is present in cancer-related and unrelated genes. The external validation conducted in women with basal breast cancer revealed that only a subset of the identified DESs in women’s tumors showed an association with progression.

## 4. Discussion

The exploration of A>I(G) RNA editing in the context of drug response mechanisms in BC has unveiled a complex landscape with implications for both understanding therapeutic mechanisms and identifying potential biomarkers. The challenge of low responsiveness to therapy requires innovative approaches to uncover novel therapeutic targets and biomarkers from new sources such as epitranscriptomics. In the context of characterizing BC cell lines, our examination reveals no significant differences in the global editing index, *ADAR1*, or *ADAR2* expression between low- and high-sensitivity groups to genotoxic drugs among BC cell lines. Remarkably, our findings align with those reported in cell lines resistant and sensitive to letrozole and doxorubicin [22]. Within our BC cell lines, a positive correlation emerged between *ADAR1* expression and the global editing index. A trend also was found in cancer cell lines [31] and in patients with acute myeloid leukemia (AML) [32]. This correlation suggests that the global editing index may only partially explain the RNA-editing activity of ADAR1, emphasizing the need for a more targeted approach focusing on specific RNA-edited sites.

We describe a comprehensive landscape of DESs associated with drug sensitivity. Recently, in a study about A>I(G) RNA editing across all available cancer cell lines, only two RNA-edited sites, Chr19:58355670 (A/G) in *ZNF587B* and Chr20:36147563 (T/C) in *BLCAP*, were associated with sensitivity to doxorubicin specifically in BC cell lines [31]. In contrast, our study delves deeper into the RNA-editing landscape of BC cell lines, offering a thorough characterization of an extensive and stringent list of DESs linked to genotoxic drug sensitivity. Because we found significant differences in the gene expression of DES-related genes per group, it suggests that gene expression alone does not explain the different drug sensitivities. Delving into those DESs with harmful functional consequences could contribute to elucidating the understanding of drug sensitivity. Previously, we discussed the impact of gene edits on genes associated with gene regulation, DNA damage response, apoptosis, and the cell cycle. We specifically focused on these dimensions.

Regarding cancer-related genes, DESs were also found in genes related to proliferation and apoptosis. Specifically, DESs within the *TP53* transcript, such as Chr17:7578442 (T/C) with the missense amino acid change Y163C, were identified across three families and associated with low sensitivity. Conversely, the Y220C amino acid change in TP53 was linked to high sensitivity to PARPis and alkylating agents, alongside low sensitivity to anthracyclines. The TP53^Y220C^ and TP53^Y163C^ mutations are cataloged as likely oncogenic in OncoKB. Interestingly, a new drug targeting the TP53^Y220C^ mutation is currently in phase 1/2 of clinical trials focusing on advanced solid tumors (ClinicalTrials.gov NCT04585750). Our analysis identified DESs within genes associated with DNA damage repair, such as *ERCC4*, *BRCA1*, and *PMS2*, and genes involved in gene expression regulation such as *NUP98* and *KMT2D*. A DES in *ERCC4,* a gene for excision repair 4, endonuclease catalytic subunit, which is part of a complex structure-specific DNA repair process, caused an E875G change, and this variant has been documented in patients with hereditary BC [33]. Also, a DES in *BRCA1* that caused a Q356R change has previously been characterized as a variant of resistance to cisplatin and olaparib, drugs of the anthracycline and PARPi families, respectively [34]. Furthermore, a DES in *PMS2*, a gene for a component of mismatch repair, that resulted in a I18V change, has been experimentally linked to the induction of microsatellite instability, a hallmark of mismatch repair-deficient cancer [35].

Additionally, our findings reveal that prioritized DESs with predicted functional damage in non-related cancer genes (according to the CGC) could be involved in drug response, such as Chr17:33430313 (T/C) in *RAD51D*, a gene involved in DDR and homologous recombination (HR), with a E253G change identified in our study, which has been reported in ovarian and breast cancer patients. This variant segregates with another variant in *RAD51D*, c.620C>T; p.S207L, with both affecting HR and providing sensitivity to PARPi [36]. In our exploration of *TPMT*, a gene involved in drug metabolism, we found a DES with potential clinical relevance, Chr6:18130918 (T/C). Notably, a significantly higher mRNA expression of *TMPT* (*p*-value = 0.015) in patients with BC who did not respond to chemotherapy compared to those who did has been demonstrated [37].

In recent publications, studies based on RNA editing have emerged, showcasing their utility in clinical contexts. Importantly, an RNA editing signature has been identified as a prognostic and predictive biomarker in gastric cancer [38], and a response to immunotherapy in lung cancer [39]. Additionally, RNA-edited sites, including the specific site in *SOCS2-AS1* at Chr12:935,429,429 (T/C) (*n* = 32), has been linked to survival probability in AML patients compared to non-edited sites (*n* = 119) [32]. Although these DESs could be relevant to drug response, we aimed to address sites with clinical significance. Therefore, we focused on those related to progression, revealing associations between certain RNA-edited sites and poor survival outcomes, despite the analysis being conducted only in those women with those DESs. Notably, we observed that hyper-editing of the *LSR* transcript (Chr19:35267377 (A/G)) with a synonymous amino acid change (LSR^519R^, agA/agG) was associated with progression of the disease. Higher mRNA *LSR* expression has been related to poor survival compared with low mRNA *LSR* expression in patients with subtype luminal A, B, and basal-like BC, and in the METABRIC cohort [40], and plays a negatively regulating tumor immunity role by inhibiting CD8+ T cells [41].

We also found that hypo-editing of Chr1:27935082 (T/C) in the 5’UTR of *SMPDL3B* has been related to poor prognosis of the disease. *SMPDL3B* is a tumor suppressor gene, which when expressed at low mRNA gene expression levels has been associated with poor prognosis in invasive ductal carcinoma [42]. Additionally, we noted that a specific DES at Chr8:38972531 (A/G) in an Alu element in *HTRA4* when hypo-edited was associated with a faster progression of the disease. *HTRA4* is a human protease implicated in various tumors, including BC, which has been associated with oncogenesis and demonstrated to promote cancer cell death and apoptosis induced by the enhanced activity of chemotherapeutic drugs in BC cell lines [43], and is a potential prognostic biomarker for BC [44]. Finally, our analysis identified that hypo-editing (Chr22:22306953 (T/C)) in *LL22NC03-80A10.6* is associated with poor PFS, remarkably, this lncRNA is enriched in tumoral tissue in papillary thyroid carcinoma [45].

These findings highlight the role of RNA editing in BC. However, the study’s limitations include non-discriminatory cell line selection based on luminal or basal subtype, or BRCA1 germline variant presence. Our analysis focused on sites with a minimum of five edited reads, possibly missing hypo-edited sites but allowing higher specificity in RNA edition detection. However, importantly, most identified sites are in non-coding regions, potentially indicating true RNA-edited sites [46,47]. Our findings underscore the potential role of RNA-edited sites as a new source of biomarkers.

## 5. Conclusions

Our study provides an epitranscriptomic perspective on drug response, with a specific emphasis on identifying a profile of DESs associated with drug sensitivity in BC cell lines that were validated in BC women patients. We focused on characterizing a subset of potential editing sites that could be involved in the progression of disease; these must be validated in vitro. Regarding the underlying mechanisms for drug response, research is needed to explore other relevant variables. Future studies should aim to integrate clinical data and pathological information with a multi-omics approach, including epitranscriptomics.

## Data Availability

The codes are deposited in https://github.com/ybernalg/RNA_editing_drugresponse_breastcancer. Additional inquiries about the codes are available from the corresponding author upon reasonable request.

## Supplementary Materials

The following supporting information can be downloaded at https://www.mdpi.com/article/10.3390/biomedicines12040728/s1: Table S1. List of differentially A>I(G) RNA-edited sites in PARPi, anthracyclines, and alkylating agents; Table S2. List of common differentially A>I(G) RNA-edited sites between PARPi, anthracyclines, and alkylating agents; Table S3. List of genes of differentially A>I(G) RNA-edited sites shared between PARPi, anthracyclines, and alkylating agents; Table S4. List of genes of common differentially A>I(G) RNA-edited sites between PARPi, anthracyclines, and alkylating agents by drug sensitivity; Table S5. Differential gene expression analysis in genes of DESs in PARPi, anthracyclines, and alkylating agents. Wilcoxon test and Benjamini–Hochberg correction; Table S6. Splicing alteration of DESs in PARPi, anthracyclines, and alkylating agents; Table S7. Pathogenic DESs associated with drug sensitivity in transcripts of unrelated cancer genes from COSMIC database; Table S8. Pathogenic DESs associated with drug sensitivity with damaging consequences predicted in transcripts of related cancer genes from COSMIC database; Table S9. Clinical and molecular characterization of 86 women with basal breast cancer from TCGA; Table S10. Survival analysis of progression-free survival in 86 women with basal breast cancer from TCGA by RNA editing level. Figure S1. Characterization of ADAR2 expression in PARPi, anthracyclines, and alkylating agents; Figure S2. Flow chart of identification of A>I(G) RNA-edited sites in selected breast cancer cell lines; Figure S3. Proportion of Alu elements in DESs by drug sensitivity in PARPi, anthracyclines, and alkylating agents; Figure S4. Standardization of the average RNA editing level in 74 DESs found in women with BC by drug sensitivity, with low sensitivity in blue, and high sensitivity in yellow, in PARPi (A), anthracyclines (B), and alkylating agents (C).

## Abbreviations

A>I(G): adenosine (A) to inosine (I) (A>I), interpreted as guanosine (G); ADAR 1: adenosine deaminase acting on RNA 1; ADAR 2: adenosine deaminase acting on RNA 2; ADAR 3: adenosine deaminase acting on RNA 3; AEI: Alu Editing Index; AML: acute myeloid leukemia; AZIN1: antizyme inhibitor 1; BC: breast cancer; BH: Benjamini–Hochberg; BLCAP: BLCAP apoptosis inducing factor; BRCA1: breast cancer gene 1; CCLE: Cancer Cell Line Encyclopedia; CGC: Cancer Gene Census; COG3: conserved oligomeric Golgi complex subunit 3; COPA: COPI coat complex subunit alpha; COSMIC: Catalogue Of Somatic Mutations In Cancer; DDR: DNA damage response; DES: differentially RNA-edited sites; DHFR: dihydrofolate reductase; dsRNA: double-stranded RNA; ERCC4: ERCC excision repair 4, endonuclease catalytic subunit; FDR: false discovery rate; GDSC1: Genomics of Drug Sensitivity in Cancer 1; GDSC2: Genomics of Drugs Sensitivity in Cancer 2; GRIA2: glutamate ionotropic receptor AMPA type subunit 2; HR: homologous recombination; HTRA4: HtrA serine peptidase 4; IC_50_: half-maximal inhibitory concentration; KMT2D: lysine-specific methyltransferase 2D; lncRNAs: long non-coding RNAs; LSR: lipolysis-stimulated lipoprotein receptor; MLE: maximum likelihood estimate; NUP98: nuclear pore complex protein Nup98-Nup96; PARPis: poly ADP-ribose polymerase inhibitors; PFS: progression-free survival; PMS2: PMS1 homolog 2, mismatch repair system component; PolyPhen: Polymorphism Phenotyping; RAD51D: RAD51 paralog D; SIFT: Sorting Intolerant From Tolerant; SNP: single nucleotide polymorphism; SMPDL3B: sphingomyelin phosphodiesterase acid-like 3B; SOCS2-AS1: suppressor of cytokine signaling 2-antisense transcript 1; TCGA: The Cancer Genome Atlas; TKIs: tyrosine kinase inhibitors; TP53: tumor protein p53; TPM: transcript per million; TPMT: thiopurine methyltransferase or thiopurine S-methyltransferase; UTR: untranslated region; VEP: Ensembl Variant Effect Predictor; ZNF587B: zinc finger protein 587B.
