# Supplementary material for: A Comprehensive Analysis of the Effect of A>I(G) RNA-Editing Sites on Genotoxic Drug Response and Progression in Breast Cancer
Source: Biomedicines. 2024 Mar 25;12(4):728. doi: 10.3390/biomedicines12040728 (PMC11048297; doi:10.3390/biomedicines12040728)
Supplement: Supplementary file 1 [file biomedicines-12-00728-s001.zip › Table S7-S9.pdf]

Table S7. Pathogenic DES associated with drug in transcripts of unrelated cancer genes from COSMIC database. SIFT (Sorting Intolerant From Tolerant), PolyPhen (Polymorphism Phenotyping), Clinical significance (ClinVar; clinical significance), Combined Annotation-Dependent Depletion (CADD) Phred score by Variant Effect Predictor.

**Chr:Position**	**Gene name**	**Drug sensitivity**	**Amino acids**	**Codons**	**SIFT**	**PolyPhen**	**ClinVar**	**CADD PHRED**
Chr4:113359801_T/C	ALPK1	HS PARPi<br>HS anthracyclines<br>HS Alkylating agents	L1117P	cTg/cCg	Deleterious (0)	Probably damaging (0.999)	benign	32
Chr 11:118971106_T/C	DPAGT1	LS PARPi<br>LS anthracyclines*<br>LS Alkylating agents	Y170C	tAt/tGt	Deleterious (0)	Probably damaging (0.997)	VUS, pathogenic	31
Chr 3:33093480_T/C	GLB1	LS_PARPi<br>LS anthracyclines<br>LS Alkylating agents	Y270C	tAt/tGt	Deleterious (0)	Probably damaging (0.997)	Likely pathogenic	29.8
Chr17:33430313_T/C	RAD51D	LS_PARPi<br>LS anthracyclines<br>LS Alkylating agents	E253G	gAg/gGg	Deleterious (0.03)	Probably damaging (0.999)	benign/likely benign, benign	28.9
Chr 12:56814403_A/G	TIMELESS	LS PARPi<br>LS anthracyclines<br>LS Alkylating agents	F1060L	Ttt/Ctt	Deleterious (0)	Probably damaging (0.999)	benign	28.7
Chr 2:70504399_A/G	PCYOX1	LS PARPi<br>LS Alkylating agents	S465G	Agt/Ggt	Deleterious (0.01)	Probably damaging (0.999)	benign	28
Chr 17:39977249_T/C	FKBP10	HS_PARPi	I436T	aTc/aCc	Deleterious (0)	Probably damaging (0.999)	Likely benign, CIP	27.9
**Chr 6:18130918_T/C**	**TPMT**	**LS PARPi<br>LS anthracyclines<br>LS Alkylating agents**	**Y240C**	**tAt/tGt**	**Deleterious (0)**	**Probably damaging (0.998)**	**Likely benign, drug response**	**27.7**
Chr 12:6458350_A/G	SCNN1A	LS PARPi<br>HS anthracyclines<br>LS Alkylating agents	W552R	Tgg/Cgg	Deleterious (0)	Probably damaging (1)	Benign/ likely benign	27.4

Chr 11:73796878_T/C	C2CD3	HS PARPi<br>LS anthracyclines<br>LS Alkylating agents	N1232S	aAt/aGt	Deleterious (0.02)	Probably damaging (0.994)	Benign/likely benign	26.8

Table S8. Pathogenic DES associated with drug sensitivity with damaging consequences predicted in transcripts of related cancer genes from COSMIC database. SIFT (Sorting Intolerant From Tolerant), PolyPhen (Polymorphism Phenotyping), Clinical significance (ClinVar; clinical significance) by Variant Effect Predictor; TSG (tumour suppressor gene) from CGC from COSMIC database.

**Chr:Position**	**Gene name**	**Drug sensitivity**	**Amino acids**	**Codon**	**SIFT**	**PolyPhen**	**ClinVar**	**Role in cancer by CGC**
Chr 11:3800205_T/C	NUP98	HS PARPi<br>HS anthracyclines<br>HS Alkylating agents	T85A	Aca/Gca	Deleterious (0.01)	Possibly damaging (0.58)	Benign	oncogene, fusion
Chr12:49428694_T/C	KMT2D	LS PARPi<br>LS anthracyclines<br>HS Alkylating agents	D3419G	gAt/gGt	Deleterious (0)	Probably damaging (0.996)	Likely benign	oncogene, TSG
Chr 16:14042077_A/G	ERCC4	LS PARPi<br>LS anthracyclines<br>LS Alkylating agents	E875G	gAa/gGa	Deleterious (0.02)	Possibly damaging (0.585)	Benign, likely benign	TSG
Chr 17:41246481_T/C	BRCA1	HS PARPi<br>HS anthracyclines	Q356R	cAg/cGg	Deleterious (0.02)	Possibly damaging (0.795)	Benign, VUS	TSG
Chr 17:7578190_T/C	TP53	LS PARPi<br>LS anthracyclines*<br>HS Alkylating agents	Y220C	tAt/tGt	Deleterious (0)	Probably damaging (0.998)	Likely pathogenic, pathogenic	oncogene, TSG, fusion
Chr17:7578442_T/C	TP53	HS PARPi<br>LS anthracyclines*<br>LS Alkylating agents	Y163C	tAc/tGc	Deleterious (0)	Probably damaging (0.999)	Likely pathogenic, pathogenic	oncogene, TSG, fusion

Chr4:106197000_A/G	TET2	HS PARPi<br><br>LS anthracyclines	H1778	cAt/cGt	Deleterious (0.01)	Possibly damaging (0.551)	Not provided	TSG
Chr7:6045634_T/C	PMS2	LS PARPi<br><br>LS anthracyclines<br>LS Alkylating agents	I18V	Att/Gtt	Deleterious (0)	Probably damaging (0.994)	Benign, VUS, benign/likely benign, likely benign, CIP	TSG

Table S9. Clinical and molecular characterization of 86 women with basal breast cancer from TCGA.

	Low-PFS (n=43)	High-PFS (n=43)	p-value
**Age at diagnosis (years)**			0.111
Mean (SD)			
Median (min, Max)	58.2 (12.4)	53.8 (13.1)	
	56 (29-84)	51 (29, 83)	
**Progression-Free survival (months)**			<0.001
Mean (SD)			
Median (min, Max)	6.49 (4.76)	96.5 (39.9)	
	7.46 (0, 13.6)	89.5 (55.6, 256)	
**TMB non-synonymous**			0.685
Mean (SD)			
Median (min, Max)	3.62 (6.13)	3.16 (4.11)	
	2.63 (0.167, 41.8)	2.27 (0.5, 28)	
**Mutation count**			0.609
Mean (SD)	107 (184)	89.8 (122)	
Median (min, Max)	78 (6, 41.8)	57 (15, 827)	
**Ethnicity category**			1
Not Hispanic or latino	36 (83.7%)	36 (83.7%)	
Hispanic or latino	0 (0%)	1 (2.3%)	
Missing	7 (16.3%)	6 (14%)	
**Ancestry**			0.629
AFR	6 (14%)	4 (9.3%)	
AFR_ADMIX	4 (9.3%)	3 (7%)	
EUR	26 (60.5%)	31 (72.1%)	
Missing	7 (16.3%)	0 (0%)	
**Race category**			0.0487
Asian	5 (11.6%)	0 (0%)	
Black or African American	11 (25.6%)	11 (25.6%)	
White	24 (55.8%)	32 (74.4%)	
Missing	3 (7%)	0 (0%)	
**Tumor type**			0.409
Infiltrating carcinoma (NOS)	1 (2.3%)	0 (0%)	
Infiltrating ductal carcinoma	37 (86%)	39 (90.7%)	
Infiltrating lobular carcinoma	1 (2.3%)	0 (0%)	

Medullary carcinoma	1 (2.3%)	1 (2.3%)	
Metaplastic carcinoma	2 (4.7%)	0 (0%)	
Other	1 (2.3%)	3 (7%)	
**Tumor stage code**			0.159
T1	1 (2.3%)	3 (7%)	
T1C	3 (7%)	9 (20.9%)	
T2	30 (69.8%)	25 (58.1%)	
T3	5 (11.6%)	4 (9.3%)	
T4	2 (4.7%)	0 (0%)	
T4B	2 (4.7%)	0 (0%)	
T4D	0 (0%)	1 (2.3%)	
TX	0 (0%)	1 (2.3%)	
**Lymph node stage code**			0.138
N0	21 (48.8%)	15 (34.9%)	
N0 (I-)	7 (16.3%)	11 (25.6%)	
N1	4 (9.3%)	5 (11.6%)	
N1A	2 (4.7%)	5 (11.6%)	
N2	4 (9.3%)	1 (2.3%)	
N3	1 (2.3%)	0 (0%)	
N3A	2 (4.7%)	0 (0%)	
N3B	1 (2.3%)	0 (0%)	
N3C	1 (2.3%)	0 (0%)	
N0 (I+)	0 (0%)	1 (2.3%)	
N1B	0 (0%)	3 (7%)	
N2A	0 (0%)	2 (4.7%)	
**Metastasis stage code**			0.359
M0	38 (88.4%)	40 (93%)	
M1	2 (4.7%)	0 (0%)	
MX	3 (7%)	3 (7%)	
