# Supplementary figures and images for: A Comprehensive Analysis of the Effect of A>I(G) RNA-Editing Sites on Genotoxic Drug Response and Progression in Breast Cancer
Source: Biomedicines. 2024 Mar 25;12(4):728. doi: 10.3390/biomedicines12040728 (PMC11048297; doi:10.3390/biomedicines12040728)

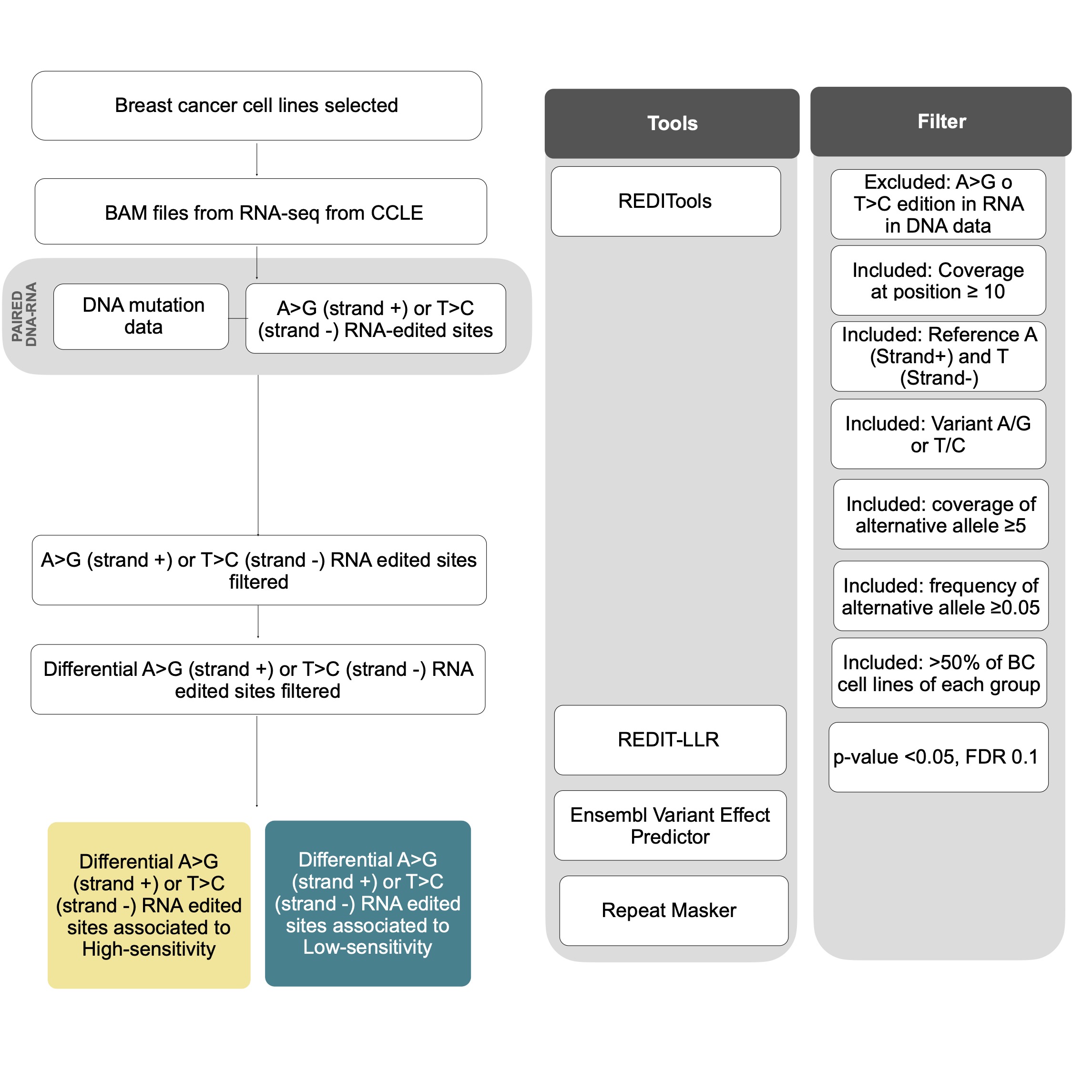

Supplement: Supplementary file 1 [file biomedicines-12-00728-s001.zip › Figure S2.jpg]
